# MRPL44 regulates lipid metabolism in metabolic dysfunction-associated steatotic liver disease through BNIP3-mediated mitophagy

**DOI:** 10.3389/fnut.2025.1662882

**Published:** 2025-10-02

**Authors:** Siqi Liu, Lianggui Xiao, Qiuchen Cheng, Qichao Liao, Yuxin Huang, Xiangling Li, Zhiwang Zhang, Yang Xiao, Zupeng Luo, Tingli Pan, Yu Sun, Chang Sun, Jiale Wang, Lin Yu, Turtushikh Damba, Batbold Batsaikhan, Xue Liang, Yunxiao Liang, Khongorzul Batchuluun, Yixing Li, Lei Zhou

**Affiliations:** ^1^Institute of Digestive Disease, Guangxi Academy of Medical Sciences, The People’s Hospital of Guangxi Zhuang Autonomous Region, Nanning, China; ^2^Guangxi Key Laboratory of Animal Breeding, Disease Control and Prevention, College of Animal Science and Technology, Guangxi University, Nanning, China; ^3^School of Pharmacy, Mongolian National University of Medical Sciences, Ulan Bator, Mongolia; ^4^Department of Internal Medicine, Institute of Medical Sciences, Mongolian National University of Medical Sciences, Ulan Bator, Mongolia; ^5^Department of Health Research, Graduate School, Mongolian National University of Medical Sciences, Ulan Bator, Mongolia; ^6^Institute of Biomedical Sciences, Mongolian National University of Medical Sciences, Ulan Bator, Mongolia

**Keywords:** fatty acid oxidation, mitophagy, mitochondrial quality, MRPL44, metabolic dysfunction-associated steatotic liver disease

## Abstract

**Objective:**

Mitophagy is a critical defense mechanism against metabolic dysfunction–associated steatotic liver disease. MRPL44, a mitochondrial ribosomal protein that regulates mitochondrial DNA-encoded gene expression, has not previously been linked to lipid metabolism.

**Methods:**

This study employed an oleic acid/palmitic acid induced HepG2 cell models and a high-fat diet fed mouse models, combined with lentivirus-mediated MRPL44 overexpression and mitophagy assays, to investigate the regulatory role of MRPL44 in the progression of metabolic dysfunction–associated steatotic liver disease.

**Results:**

Our findings demonstrated that MRPL44 alleviates lipid metabolic disorders induced by high-fat diet through the mitophagy pathway. Specifically, in oleic acid/palmitic acid-stimulated HepG2 cells, overexpression of MRPL44 reduced intracellular triglyceride accumulation and enhanced fatty acid oxidation. Moreover, liver-specific overexpression of MRPL44 in mice attenuated high-fat diet induced hepatic lipid deposition. Mechanistically, MRPL44 activated the BNIP3-dependent mitophagy pathway, promoted mitochondrial biogenesis, and mitigated mitochondrial damage, ultimately reducing lipid accumulation in hepatocytes.

**Conclusion:**

This study identifies MRPL44 as a novel regulator of lipid metabolism and a potential therapeutic target for metabolic dysfunction–associated steatotic liver disease.

## Graphical abstract

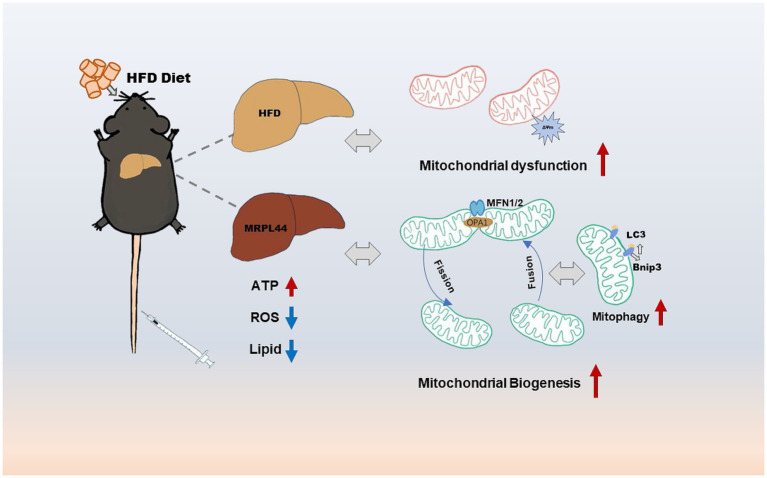

## Introduction

Metabolic dysfunction-associated steatotic liver disease (MASLD), formerly known as non-alcoholic fatty liver disease (NAFLD), represents the most common chronic liver disorder worldwide. Its redefinition underscores the strong link between hepatic steatosis and metabolic abnormalities, including obesity, type 2 diabetes, dyslipidemia, and insulin resistance (1). MASLD is characterized by excessive lipid accumulation in hepatocytes and disease progression driven by oxidative stress, inflammation, and hepatocellular injury, ultimately leading to liver fibrosis and cirrhosis (2). Mounting evidence indicates that lipid metabolic imbalance, mitochondrial dysfunction, oxidative stress, and chronic inflammation constitute the central mechanisms underlying MASLD pathogenesis (3). Mitochondria, as the key organelles for ATP production, reactive oxygen species (ROS) generation, and lipid metabolism, are indispensable for maintaining hepatic metabolic homeostasis (4). Impairment of mitochondrial oxidative phosphorylation diminishes ATP synthesis, inhibits mitochondrial biogenesis, and triggers mitochondrial dysfunction, thereby accelerating MASLD progression (5). Therefore, strategies aimed at preserving mitochondrial quality, and enhancing mitochondrial biogenesis are expected to improve lipid metabolism, and offer potential therapeutic avenues for MASLD.

Mitochondrial quality control includes biogenesis, mitochondrial fission and fusion, mitophagy (6). When mitochondria are subjected to external stress or oxidative damage, they release excessive ROS, exacerbating mitochondrial oxidative stress. To maintain normal mitochondrial function, damaged mitochondria can be repaired by fusion or fission with healthy mitochondria. Additionally, damaged mitochondria can be removed by mitophagy (7). Mitophagy is a targeted mechanism for clearing damaged mitochondria. It involves key proteins, including BNIP3, PARK2, PINK1, and LC3 (8). These proteins localized to the mitochondrial membrane, thereby facilitating the formation of autophagosomes and the removal of damaged mitochondria. BNIP3, a BH3-only protein of the BCL2 family, is predominantly localized to the outer mitochondrial membrane, where it participates in cellular regulation. Furthermore, BNIP3 serves as a mitophagy receptor by directly interacting with LC3, promoting the clearance of damaged mitochondria (9). Studies have demonstrated that mitophagy is essential for maintaining the normal physiological function of mitochondria, and also plays a key role in the regulation of several metabolic diseases (5). Activation of mitophagy protects mitochondria and liver cells from fatty diet-induced steatosis, thereby reducing liver damage (10).

Mitochondrial ribosomal proteins (MRPs) are complexes composed of mitochondrial ribosome RNA, proteins that significantly influence the structural, and functional integrity of mitochondrial complexes (11). The mammalian genome contains over 80 MRP genes, however, despite most of these genes being expressed in the body, MRP proteins exhibit low homology and share few common characteristics (12). This implies that various MRPs play distinct and crucial roles in tissues or cells. Most studies have established a connection between MRPs and mitochondrial diseases, prognostic biomarkers of various cancers, and/or therapeutic targets (13, 14). In our preliminary work, siRNA library screening suggested that MRPL44 expression may be negatively associated with lipid metabolism. MRPL44 is a mitochondrial ribosomal protein localized to the mitochondrial matrix. It plays an essential role in regulating the expression of mitochondrial DNA (mtDNA)-coding genes, mainly affecting mitochondrial ATP synthesis and cellular respiration (15). However, the effect of MRPL44 on liver lipid metabolism and mitochondrial mass has not been reported. The goal of this study is to investigate the role of MRPL44 in MASLD, and determine whether it improves mitochondrial function by affecting mitophagy. This may provide a new approach for the treatment of MASLD.

## Materials and methods

### Animals and treatment

Five-week-old male C57BL/6 mice were purchased from Guangxi Medical University (Guangxi, China) and used to establish the NAFLD model. MRPL44 transgenic mice were administered adeno-associated virus serotype 8 (AAV8), AAV8-CMV-MRPL44-ZsGreen via tail vein injection, while the control group received AAV8-CMV-ZsGreen. All animals were maintained under controlled conditions (23 ± 2 °C, 12 h light/dark cycle). At 8 weeks, mice were randomized into four groups and fed either a normal diet (ND) or high-fat diet (HFD) for 12 weeks, following our previously established protocol (16): (1) ND group: fed a ND and injected with AAV8-CMV-ZsGreen; (2) ND-MRPL44 group: fed a ND and injected with AAV8-CMV-MRPL44-ZsGreen; (3) HFD group: fed a HFD and injected with AAV8-CMV-ZsGreen; and (4) HFD-MRPL44 group: fed a HFD and injected with AAV8-CMV-MRPL44-ZsGreen. All animal protocols were approved by the Animal Ethics Committee of Guangxi University (GXU-2021-160).

### Body composition, micro-CT, and metabolic analysis

Lean mass and fat mass of mice were analysed using a Niumag nuclear magnetic resonance (NMR) analyser (QMR23-060H-I, Niuma, Suzhou, China). Whole-body scans were performed with micro-CT (X-ray Safety Report Skyscan 1278, Bruker, Germany). Metabolic parameters including oxygen consumption, CO₂ production, energy expenditure, respiratory exchange ratio, and activity were assessed in metabolic cages (17).

### Cell culture and transfections

HepG2 cells were maintained in Dulbecco’s modified Eagle’s medium (DMEM) (Gibco, Beijing, China) containing 1% penicillin-streptomycin, and 10% fetal bovine serum (BI, Guangzhou, China) at 37 °C and 5% CO_2_. DMEM contains 4.5 g/L glucose, L-glutamine and sodium pyruvate. Cells were seeded in 24-well plates at a ratio of approximately 1–2 × 10^5^ cells/mL per well, and treated with oleic acid/palmitic acid (OA/PA, 2:1) for 24 h. The final concentration of OA was 200 μM and that of PA was 100 μM.

### Plasmids and siRNA

Human MRPL44 (NM_022915.5) was cloned into the pcDNA3.1(−) vector. The mt-Keima plasmid was purchased from HonorGene (HonorGene, Changsha, China). siRNA library was purchased from Guangzhou RuiBo (Guangzhou RiboBio Co., Ltd., Guangzhou, China), and each siRNA contained three sequences. Negative control was purchased from Sangon Biotech (Sangon Biotech Co., Ltd., Shanghai, China). Three siMRPL44 sequences and negative control sequences have been listed in Supplementary Table S1. Transient transfections of plasmids or siRNAs were conducted with Hieff Trans^®^ Liposomal Transfection Reagent (Yeasen Biotechnology, Shanghai, China) following manufacturer conditions.

### Biochemical assays

The Triglyceride Assay Kit and Glycerol Assay Kit (Nanjing Jiancheng Bioengineering Institute, Nanjing, China) were used to assess the glycerol and triglyceride concentration. Fatty acid uptake assay was performed using the Fatty Acid Uptake Assay Kit. Lipase (LPS) activity was measured using a commercial kit (Lipase Assay Kit, Nanjing Jiancheng Bioengineering Institute, Nanjing, China). ATP content was measured using a commercial kit (ATP Assay Kit, Beyotime Biotechnology, Shanghai, China). All studies were then carried out in accordance with the manufacturer’s guidelines while quantifying protein concentrations. For each experiment, the BCA Protein Quantification Assay Kit (Beyotime Biotechnology, Shanghai, China) was used to measure the protein concentrations. The intracellular lipid deposition distribution was observed by oil red O staining, and the Oil Red O working solution was prepared in a ratio of Oil Red O storage solution to water of 3:2. Images were obtained under an inverted microscope (IX53; Olympus Corporation, Tokyo, Japan).

### RNA extraction and quantitative PCR

RNA was extracted with Trizol and the RNA concentration were determined using a microplate reader. To generate cDNA, 1 μg of RNA was reverse transcribed using the M-MLV enzyme and the random primer OLIGODT18 according to manufacturer’s instructions. For gene expression analysis, we performed quantitative real-time polymerase chain reaction (qPCR) of cDNA per sample using the GenStar commercial kit (2× RealStar Green Fast Mixture; Takara Bio, Japan). The expression of mRNA was normalized to expression of β-actin. The primer sequences were listed in Supplementary Table S2. Each experiment was repeated at least three times.

### Immunofluorescent and reactive oxygen species staining

For immunofluorescence staining, HepG2 were washed with PBS and fixed in 4% paraformaldehyde (Solarbio, Beijing, China). After permeabilization in 0.5% Triton X-100 and blocked with 5% BSA (Solarbio, Beijing, China). Cells were incubated with primary antibodies at 4 °C overnight. Subsequently, the samples were washed with PBS and incubated secondary antibody with fluorescent label. The primary antibody used in immunofluorescence was: rabbit anti-LC3 (T55992S, Abmart, 1:500). Reactive Oxygen Species Detection Kit from Beyotime Biotechnology to observe and analyze intracellular ROS production. Images were captured using an inverted fluorescence microscope (IX53; Olympus Corporation, Tokyo, Japan), and the fluorescence values of ROS were quantified using a microplate reader (Infinity M200 PRO, Tecan, Switzerland).

### Oxidative stress assays

Commercial chemical assays purchased from Beyotime Biotechnology were used to determine the content of glutathione peroxidase (GPx), superoxide dismutase (SOD), and malondialdehyde (MDA). The obtained supernatant was used to measure changes in these markers of oxidative stress following the manufacturer’s instructions.

### Mitochondrial function

MitoTracker^®^ Green probe (Yeasen Biotechnology, Shanghai, China) was used for mitochondrial staining. DNA was isolated from HepG2 cells using PCR lysis buffer (18), and the relative content of mitochondrial DNA and nuclear DNA was determined by quantitative PCR. The mitochondrial membrane potential assay kit with JC-1 (Beyotime Biotechnology, Shanghai, China) was used to detect mitochondrial membrane potential. Mitophagy levels were evaluated based on the colocalization of mitochondria and lysosomes, as well as the expression of the mt-Keima plasmid. LysoTracker Red DND-99 (Yeasen Biotechnology, Shanghai, China) was used to label intracellular lysosomes with red fluorescence.

### Oxygen consumption rates

Oxygen consumption rate (OCR) was measured in HepG2 cells using a Seahorse XF24 Metabolic Extracellular Flux Analyzer (Seahorse Biosciences, North Billerica, MA, United States). Oligomycin (1 μM), Trifluoromethoxy carbonylcyanide phenylhydrazone (FCCP, 1 μM), and rotenone/antimycin A (ROT/AA, 0.5 μM) were sequentially injected. Values were normalized against protein concentration.

### Protein interaction and validation assays

The amino acid sequences of candidate proteins MRPL44 (Q9H9J2) and BNIP3 (Q12983) were obtained from the Universal Protein Resource (Uniprot) database, and analyzed using AlphaFold3 for structure prediction (19). Protein–protein docking was performed with the HDOCK server, and structural models were visualized in PyMOL (20, 21). For experimental validation, HepG2 cell lysates were subjected to co-immunoprecipitation using MRPL44 antibody or IgG control, followed by pulldown with Protein A/G beads. The precipitated proteins were analyzed by SDS-PAGE. In addition, protein expression levels of MRPL44, BNIP3, LC3, and OXPHOS components were assessed by Western blotting. The primary antibodies used were as follows: rabbit anti-MRPL44 (16394-1-AP, Proteintech, 1:1000), mouse anti-Total OXPHOS Rodent WB antibody Cocktail (ab110413, Abcam, 1:2000), mouse anti-Membrane Integrity WB antibody Cocktail (ab110414, Abcam, 1:2000), rabbit anti-LC3 (T55992S, Abmart, 1:1000), mouse anti-BNIP3 (Cat No. 68091-1, Proteintech, 1:5000), rabbit anti-β-actin (AF5012, Beyotime Biotechnology, 1:1000).

### Statistical analysis

All experiments included at least three biological replicates. Statistical analyses between different groups were performed using the Student’s *t* test or one-way ANOVA (GraphPad Software). The data are presented as the mean ± SEM and the level of significance is indicated as follows: ^*^p < 0.05 and ^**^p < 0.01.

## Result

### MRPL44 reduces HepG2 intracellular triglyceride content

**Figure 1:**
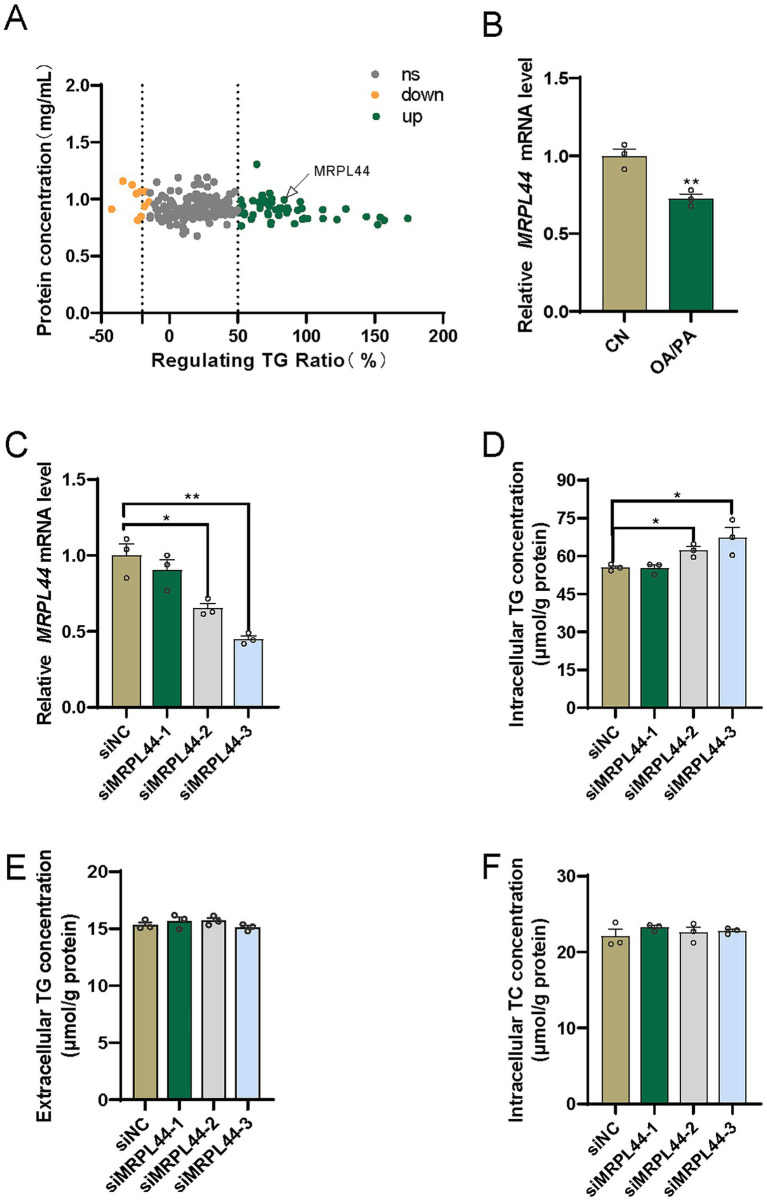
Effect of siMRPL44 on the intracellular triglyceride levels of HepG2. **(A)** siRNA library screening. **(B)** MRPL44 mRNA expression levels in NC and OAPA-treated HepG2 cells. **(C)** The expression of MRPL44 mRNA levels in OA/PA treated HepG2 cells. **(D)** Intracellular TG content in OA/PA treated HepG2 cells. **(E)** Extracellular TG content in OA/PA treated HepG2 cells. **(F)** Intracellular TC content in OA/PA treated HepG2 cells. Data are represented as means ± SEM. ^*^p < 0.05 and ^**^p < 0.01.

To identify putative genes linked to lipid metabolism, we purchased siRNA libraries and performed screening. Each target gene was represented by three siRNA sequences, which were pooled and co-transfected into HepG2 cells. After OA/PA treatment, triglyceride (TG) levels were measured and normalized to the negative control group (siNC). The data presented in Figure 1A, suggest that MRPL44 may be involved in lipid metabolism. The mRNA level of MRPL44 was examined in OA/PA-treated HepG2 cells. We found that OA/PA treatment significantly downregulated MRPL44 expression by 28% (*p* = 0.0075; Figure 1B). Subsequently, we designed three different siMRPL44 constructs to inhibit MRPL44, and their inhibition efficiencies in cells are shown in Figure 1C. Among them, siMRPL44-3 achieved the strongest knockdown effect, reducing MRPL44 expression by 55% (*p* = 0.0023). The results showed that siMRPL44 significantly increased the intracellular TG content of HepG2 cells (Figure 1D), with no significant effect on extracellular TG content (Figure 1E) or intracellular total cholesterol (TC) content (Figure 1F).

**Figure 2:**
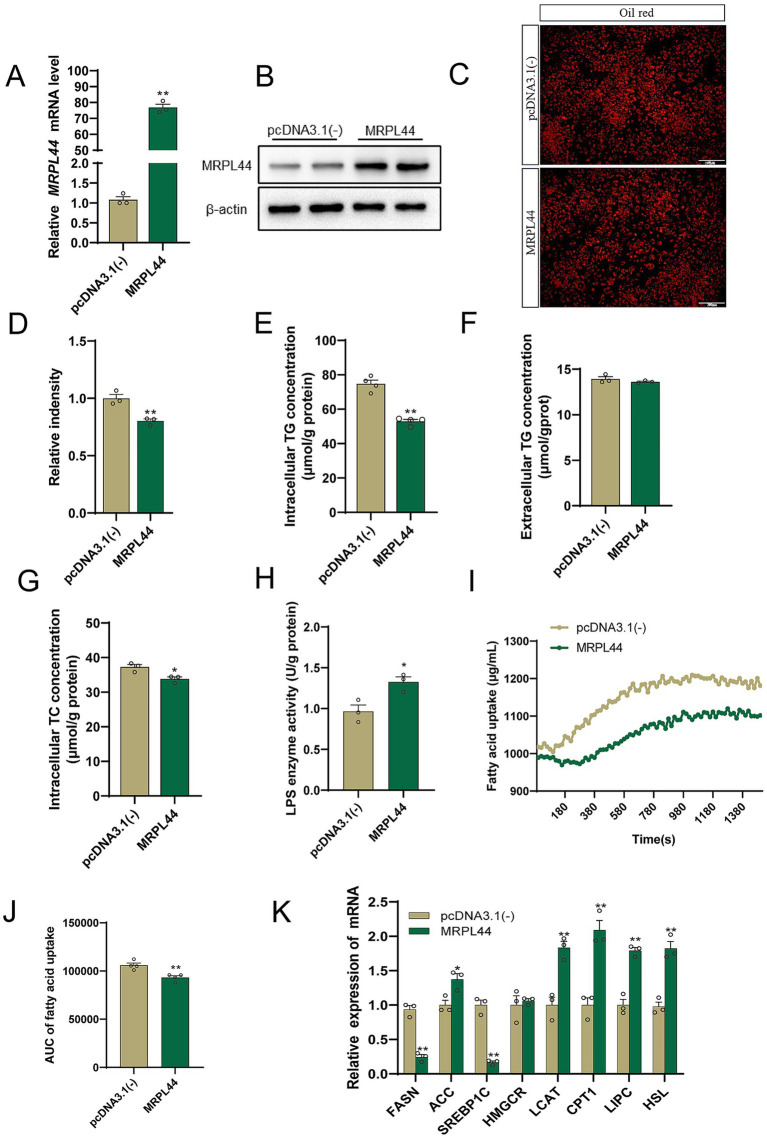
Effect of MRPL44 overexpression on lipid metabolism in OA/PA treated HepG2 cells. **(A)** The expression of MRPL44 mRNA levels. **(B)** The expression of MRPL44 protein levels. **(C)** Oil red O staining. **(D)** The quantification results from figure **C**. **(E)** Intracellular TG content in HepG2 cells. **(F)** Extracellular TG content in HepG2 cells. **(G)** Intracellular TC content in HepG2 cells. **(H)** LPS enzyme activity. **(I)** Fatty acid uptake capacity. **(J)** The quantification results from figure **I**. **(K)** Lipid metabolism-related genes mRNA expression levels. Data are represented as means ± SEM. ^*^p < 0.05 and *p* < 0.01.

To further investigate the role of MRPL44 in lipid metabolism, we overexpressed MRPL44 in HepG2 cells. Transfection of MRPL44 significantly upregulated MRPL44 expression compared with the pcDNA3.1(−) group. mRNA levels increased by approximately 75-fold (*p* < 0.001; Figure 2A), and protein levels were elevated by about 2-fold (*p* < 0.001; Supplementary Figure S1A), as confirmed in Figure 2B. Oil Red O staining demonstrated that MRPL44 overexpression reduced intracellular lipid accumulation (Figure 2C). Quantification revealed a 20% decrease in neutral lipid staining compared with the pcDNA3.1(−) group (*p* = 0.0073; Figure 2D). The intracellular TG and TC determination results showed that MRPL44 significantly reduced intracellular TG levels by 29% (*p* < 0.001, Figure 2E), with no significant effect on extracellular TG levels (Figure 2F). Moreover, MRPL44 reduced the intracellular TC levels (Figure 2G).

To explore how MRPL44 is involved in lipid deposition, we measured lipase content and fatty acid uptake capacity. MRPL44 markedly increased lipase content (Figure 2H) and significantly reduced the uptake of fatty acid (Figures 2I,J). Meanwhile, we found that MRPL44 significantly decreased the mRNA expression levels of genes associated with fatty acid synthesis (*FASN, SREBP1C*) while significantly increased the levels of mRNA expression of genes associated with fatty acid hydrolysis (*LCAT, CPT1, LIPC, HSL*) (Figure 2K). Based on these findings, we speculate that MRPL44 might inhibit lipid accumulation by encouraging fatty acid hydrolysis.

### MRPL44 enhances mitochondrial quality and function

**Figure 3:**
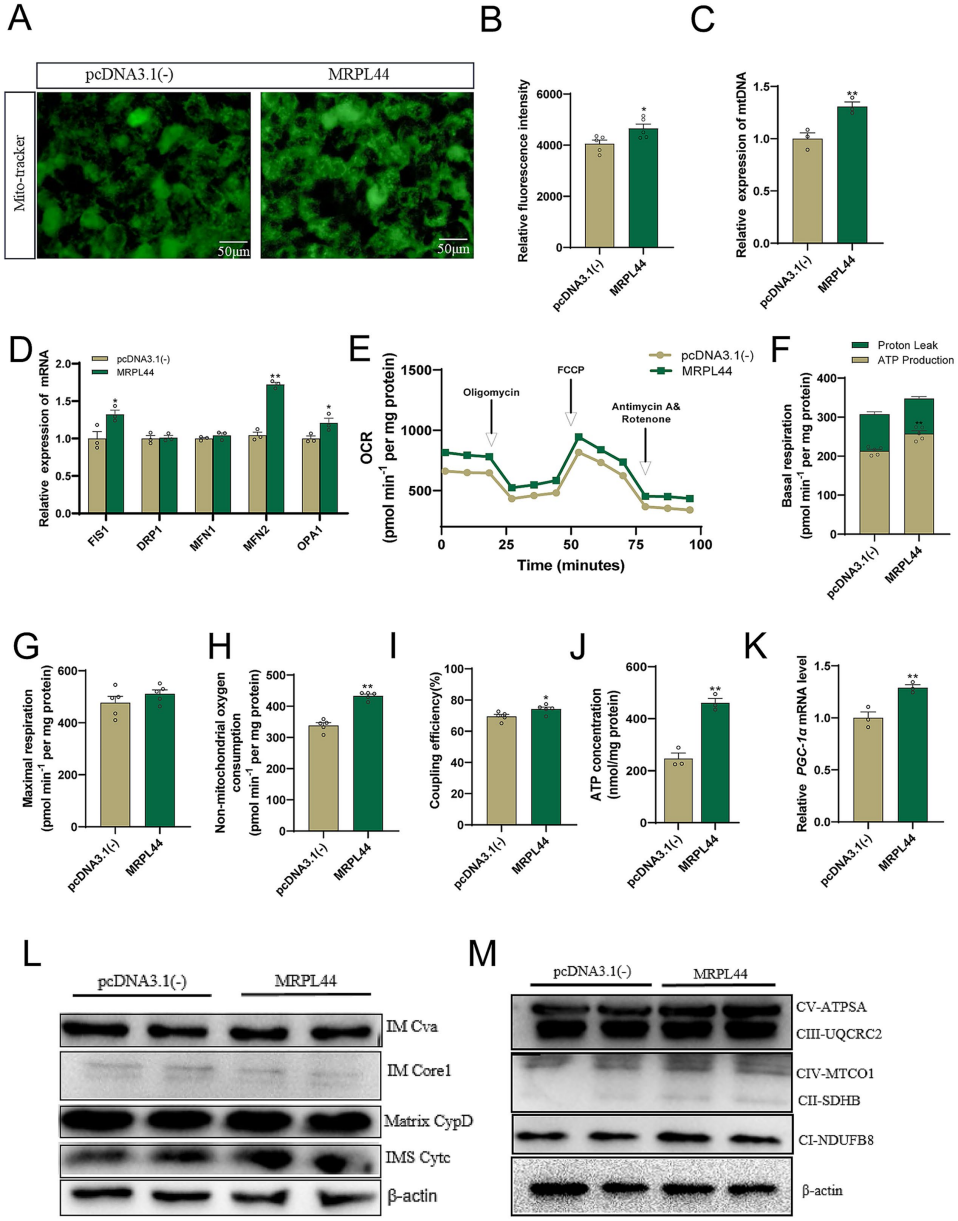
Effect of MRPL44 overexpression on mitochondria in OA/PA treated HepG2 cells. **(A)** Representative fluorescence microscopy images of mitochondria in HepG2 cells transfected with either pcDNA3.1 (−) or MRPL44 plasmids. Mito Tracker staining was used to visualize mitochondria. Scale bar: 50 μm. **(B)** Quantification of mitochondrial fluorescence probe intensity. **(C)** Relative expression levels of mitochondrial DNA. **(D)** Mitochondrial fission and fusion-related genes mRNA expression levels. **(E)** OCR. **(F)** Mitochondrial basal respiration. **(G)** Maximum respiration. **(H)** Non-mitochondrial respiration. **(I)** Coupling efficiency. **(J)** ATP production. **(K)** The expression of the PGC1-α mRNA levels. **(L)** Mitochondrial membrane-associated protein expression levels. **(M)** Mitochondrial oxidative phosphorylation-related protein expression levels. Data are represented as means ± SEM. ^*^p < 0.05 and ^**^p < 0.01.

Lipid metabolism and mitochondrial function are tightly related. To investigate the impact of MRPL44 on mitochondrial function, we performed several assays. The results of the mitochondrial fluorescent probe assay indicated that MRPL44 transfection significantly increased mitochondrial activity (Figures 3A,B), and elevated mtDNA levels by approximately 30% (*p* = 0.0126, Figure 3C). Quantitative analysis of genes associated with mitochondrial fusion, fission revealed that MRPL44 upregulated mRNA expression of the fission -related gene *FIS1* by 0.32-fold (*p* = 0.0419), and the fusion-related genes *MFN2* by 0.68-fold (*p* < 0.001), *OPA1* by 0.21-fold (*p* = 0.014) (Figure 3D). To further explore the effects of MRPL44 overexpression on mitochondrial function, we conducted a mitochondrial stress test (Figure 3E). The results demonstrated that MRPL44 enhanced basal cellular respiratory capacity, mainly accompanied by increased ATP production by 20.8% (*p* < 0.001, Figure 3F). Additionally, the maximal respiratory capacity in the MRPL44 group was slightly higher than in the pcDNA3.1 (−) group (Figure 3G), though the difference was modest. Notably, MRPL44 significantly elevated non-mitochondrial respiration by 0.28-fold (*p* < 0.001; Figure 3H), improved the coupling efficiency of the electron transport chain (Figure 3I). Consistent with these findings, intracellular ATP measurements revealed a 1.76-fold increase in ATP levels in cells overexpressing MRPL44 cells (*p* = 0.0015; Figure 3J). Moreover, we observed a 0.28-fold upregulation of *PGC-1α* mRNA, a key regulator of mitochondrial biogenesis and respiration, in MRPL44 group (*p* = 0.01; Figure 3K). We then examined the levels of mitochondrial membrane-related proteins and oxidative phosphorylation-related proteins. MRPL44 modestly upregulated the expression of several mitochondrial membrane proteins and proteins involved in oxidative phosphorylation (Figures 3L,M; Supplementary Figures S1B,C). Therefore, we propose that MRPL44 aids in increasing mitochondrial mass and maintaining mitochondrial function.

### MRPL44 attenuates oxidative stress in HepG2 cells

**Figure 4:**
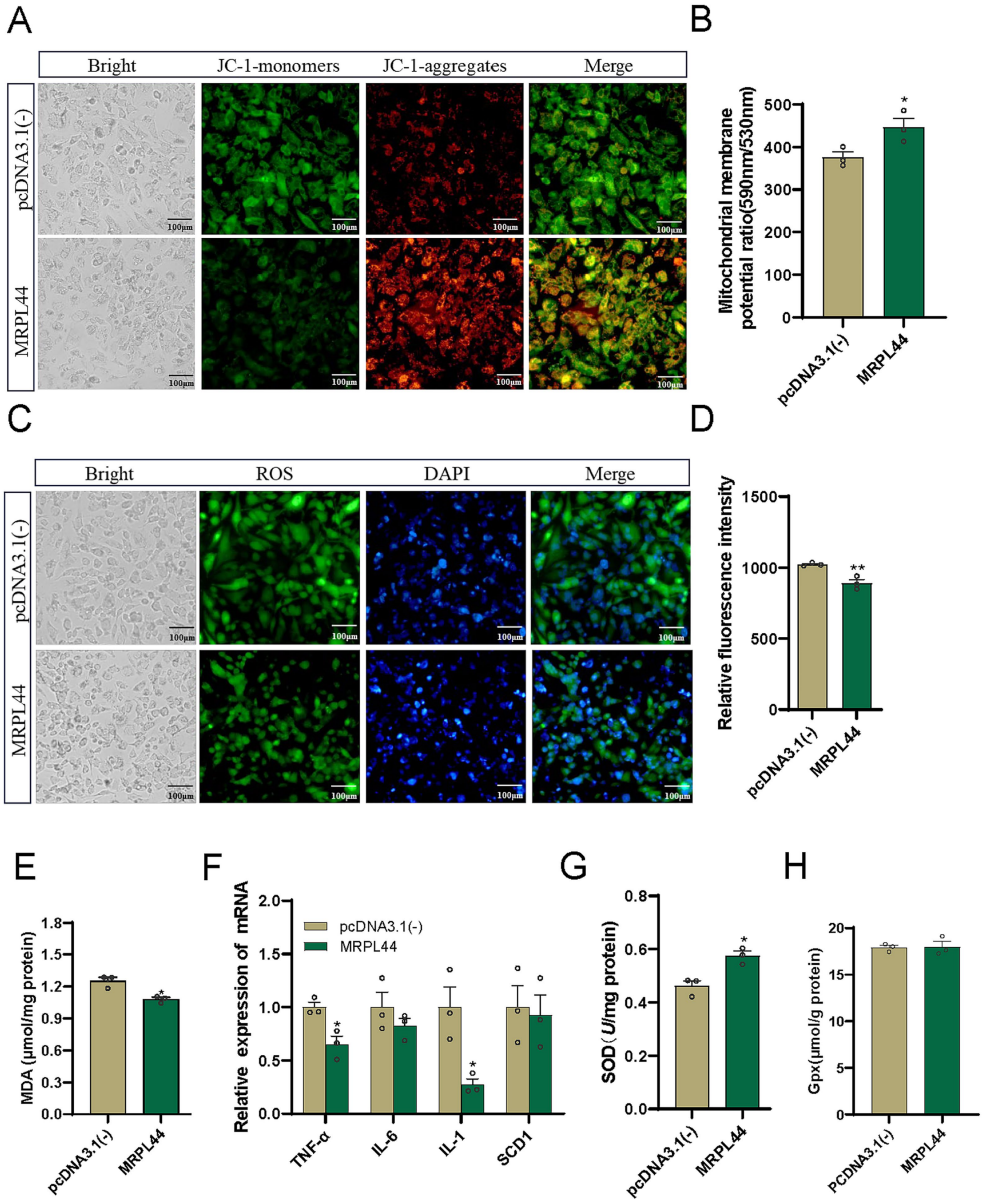
Effect of MRPL44 overexpression on oxidative stress. **(A)** Mitochondrial membrane potential in CN and OA/PA-treated HepG2 cells. Scale bar: 100 μm. **(B)** Quantification of the mitochondrial membrane potential ratio (red/green fluorescence). **(C)** Reactive oxygen staining and reactive oxygen fluorescence levels in OA/PA treated HepG2 cells. Scale bar: 100 μm. **(D)** Quantification of relative ROS fluorescence intensity. **(E)** MDA content in OA/PA treated HepG2 cells. **(F)** Oxidative stress related gene mRNA expression levels in OA/PA treated HepG2 cells. **(G)** SOD enzyme activity levels in OA/PA treated HepG2 cells. **(H)** Gpx enzyme activity levels in OA/PA treated HepG2 cells. Data are represented as means ± SEM. ^*^p < 0.05 and ^**^p < 0.01.

Lipid peroxidation causes oxidative damage in mitochondria, mitochondrial damage lowers the membrane potential of the mitochondria, and accumulates ROS. We found that MRPL44 restored the OA/PA-induced decline in mitochondrial membrane potential, as reflected by a 19% increase compared with the OA/PA group (*p* = 0.046; Figures 4A,B). MRPL44 effectively reduced intracellular ROS levels by 13% (*p* = 0.008; Figures 4C,D), decreased the content of the peroxidation product MDA by 13.7% (*p* = 0.012; Figure 4E). MRPL44 downregulated the mRNA expression of the proinflammatory cytokines TNF-α by 35% (*p* = 0.018) and IL-1 by 72% (*p* = 0.022; Figure 4F). Subsequently, MRPL44 significantly enhanced the activity of the antioxidant enzyme SOD by 24.6% (*p* = 0.013; Figure 4G), whereas no significant change was observed in GPx activity (Figure 4H). These results suggest that MRPL44 may exert protective effects against oxidative stress predominantly via the SOD pathway.

### MRPL44 improves mitophagy

**Figure 5:**
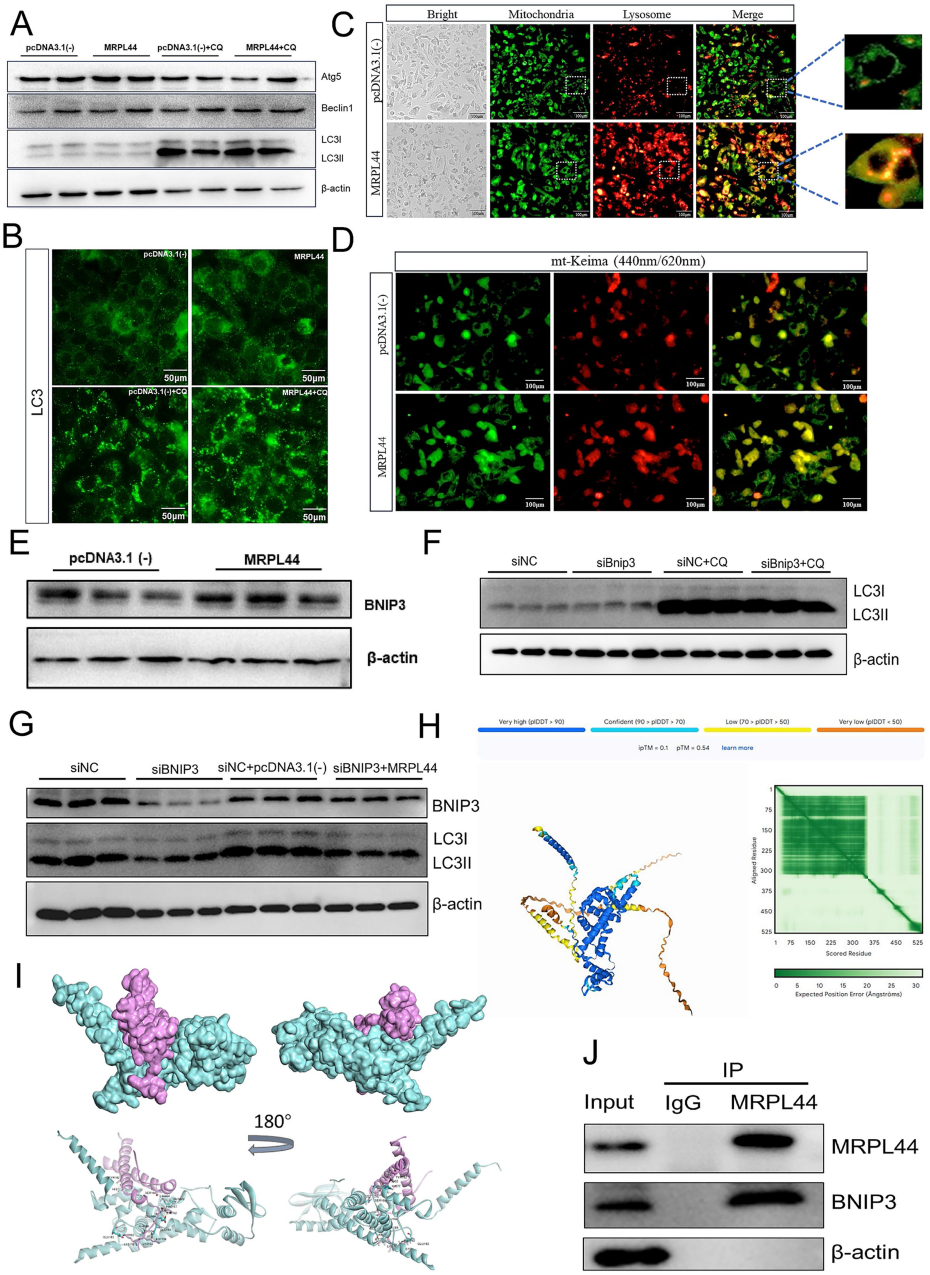
Effect of MRPL44 overexpression on autophagic flux in OA/PA treated HepG2 cells. **(A)** Western blot analysis of autophagy-related proteins (Atg5, Beclin1, LC3I, and LC3II) in cells transfected with pcDNA3.1(−) or MRPL44, with or without CQ treatment. β-actin was used as a loading control. **(B)** Immunofluorescence staining of LC3. Scale bar: 50 μm. **(C)** Representative fluorescence images showing colocalization of mitochondria (green) and lysosomes (red). Merged images show the colocalization (orange) of mitochondria and lysosomes, with zoomed regions highlighting colocalized structures. Scale bar: 100 μm. **(D)** Fluorescence microscopy of mt-Keima in cells transfected with pcDNA3.1(−) or MRPL44. The pH-dependent fluorescence of mt-Keima was used to assess mitophagy. Green fluorescence indicates neutral pH, while red fluorescence indicates acidic pH. Scale bar: 100 μm. **(E)** Western blot analysis of BNIP3 protein expression in cells. **(F)** Western blot analysis of LC3I and LC3II in cells treated with siNC or siBNIP3, with or without CQ. **(G)** Western blot analysis of BNIP3 and LC3 expression in cells treated with siNC, siBNIP3, or siBNIP3 combined with pcDNA3.1(−) or MRPL44. **(H)** Structural prediction of the MRPL44-BNIP3 complex by AlphaFold3. **(I)** Protein docking between MRPL44 (cyan) and BNIP3 (purple). **(J)** Co-immunoprecipitation (IP) assay demonstrating the interaction between MRPL44 and BNIP3. Input, total protein; control IgG, immunoprecipitated protein using negative control rabbit IgG antibody; MRPL44, immunoprecipitated protein using MRPL44 antibody; BNIP3, immunoprecipitated protein using BNIP3 antibody. Data are represented as means ± SEM. ^*^p < 0.05 and ^**^p < 0.01.

Mitophagy plays a critical role in maintaining intermediary metabolism by serving as one of the most effective mechanisms for clearing damaged mitochondria. To investigate the impact of MRPL44 on mitophagy, we first measured the expression of autophagy-related genes. MRPL44 significantly reduced LC3 I mRNA levels while markedly increasing Atg5 mRNA levels (Supplementary Figure S2A). We then used the autophagy inhibitor chloroquine (CQ, 50 μM) to further explore the effects of MRPL44 on autophagy. Western blot analysis of autophagy-related proteins revealed that MRPL44 transfection resulted in increased LC3 II protein expression, decreased LC3 I protein expression compared to the control group. Following CQ treatment, the accumulation of LC3 II protein was further enhanced (Figure 5A; Supplementary Figure S2B). Similarly, immunofluorescence staining of LC3 showed increased fluorescence intensity of LC3 protein after MRPL44 transfection, with a further increase after CQ treatment (Figure 5B; Supplementary Figure S2C).

Mitophagy is closely associated with high levels of lysosomal activity. Using fluorescence probes to label mitochondria and lysosomes, we observed that the MRPL44 group exhibited a greater number of autolysosomes compared to the pcDNA3.1 (−) group (Figure 5C). To assess MRPL44 impact on mitophagy, we used the mt-Keima plasmid. MRPL44 overexpression exhibited a stronger red fluorescence signal compared to the pcDNA3.1(−) group, indicating that mitochondria were in an acidic environment and showing higher mitophagy levels (Figure 5D). These results suggest that MRPL44 enhances mitophagy by promoting the clearance of damaged mitochondria.

### MRPL44 enhances BNIP3-mediated mitophagy

BNIP3, as a mitophagy receptor, plays a crucial role in maintaining mitochondrial homeostasis. We found that overexpression of MRPL44 significantly upregulated BNIP3 expression, with mRNA levels increased by 48% (*p* = 0.045; Figure 5E), and protein expression consistently elevated as shown in Supplementary Figures S2D,E. Inhibition of BNIP3 expression impaired autophagosome formation, leading to a reduction in LC3-II protein levels (Figure 5F; Supplementary Figure S2F). When siBNIP3 was co-transfected with MRPL44, the reduction in LC3-II expression caused by siBNIP3 was rescued (Figure 5G; Supplementary Figures S2G, S3A,B). Mitochondrial-lysosomal staining and co-localization analysis revealed that the siBNIP3 + pcDNA3.1(−) treatment group exhibited weaker mitochondrial and lysosomal fluorescence signals, whereas the siNC + MRPL44 group showed significantly enhanced co-localization between mitochondria and lysosomes. The siBNIP3 + MRPL44 treatment group partially restored this co-localization, suggesting that MRPL44 exerts a compensatory effect in the absence of BNIP3 (Supplementary Figure S3C). Furthermore, compared to the siNC group, siBNIP3 significantly increased intracellular TG levels in HepG2 cells. Co-transfection with MRPL44 alleviated the TG accumulation induced by siBNIP3 (Supplementary Figures S3E,F). These findings suggest that MRPL44 may influence BNIP3-mediated LC3 protein dynamics, thereby affecting mitophagy in HepG2 cells.

To investigate the structural basis underlying the interaction between MRPL44 and BNIP3, we utilized AlphaFold3 to predict the structure of the protein complex. The predicted model is presented in Figure 5H (the protein nucleic acid complex structure were predicted by Hefei Keiing Biotechnoloay Co., Ltd.), with per-residue confidence levels indicated by the predicted local distance difference test (pLDDT) scores. The predicted template modeling score (pTM) and the interface pTM score (ipTM) were 0.54 and 0.10, respectively. The sum of these scores (ipTM + pTM > 0.5) suggests a potential interaction between the two proteins. Additionally, the predicted aligned error (PAE) map demonstrated high intra-domain positional accuracy, further supporting the reliability of the individual structural predictions. Subsequent protein–protein docking analysis revealed that the two proteins, MRPL44 (cyan) and BNIP3 (purple) form a stable docking interface (Figure 5I). Structural displays from different angles indicate that the two have tight spatial matching and potential interaction regions on the surface, suggesting that MRPL44 and BNIP3 may have direct physical binding, providing a structural basis for their regulation of mitophagy. Magnified views of the docking model highlighted several key intermolecular contacts, residues such as ALA188, TYR182, and PHE157 of BNIP3 form hydrogen bonds and electrostatic interactions with GLU163, LYS153, and GLU183 of MRPL44 (Supplementary Figure S3D). Furthermore, co-immunoprecipitation assays confirmed the physical association between MRPL44 and BNIP3 (Figure 5J). These results suggest that MRPL44 may activate the mitochondrial autophagy process through the BNIP3-LC3 axis.

### Liver-specific overexpression of MRPL44 ameliorates hepatic lipid deposition

**Figure 6:**
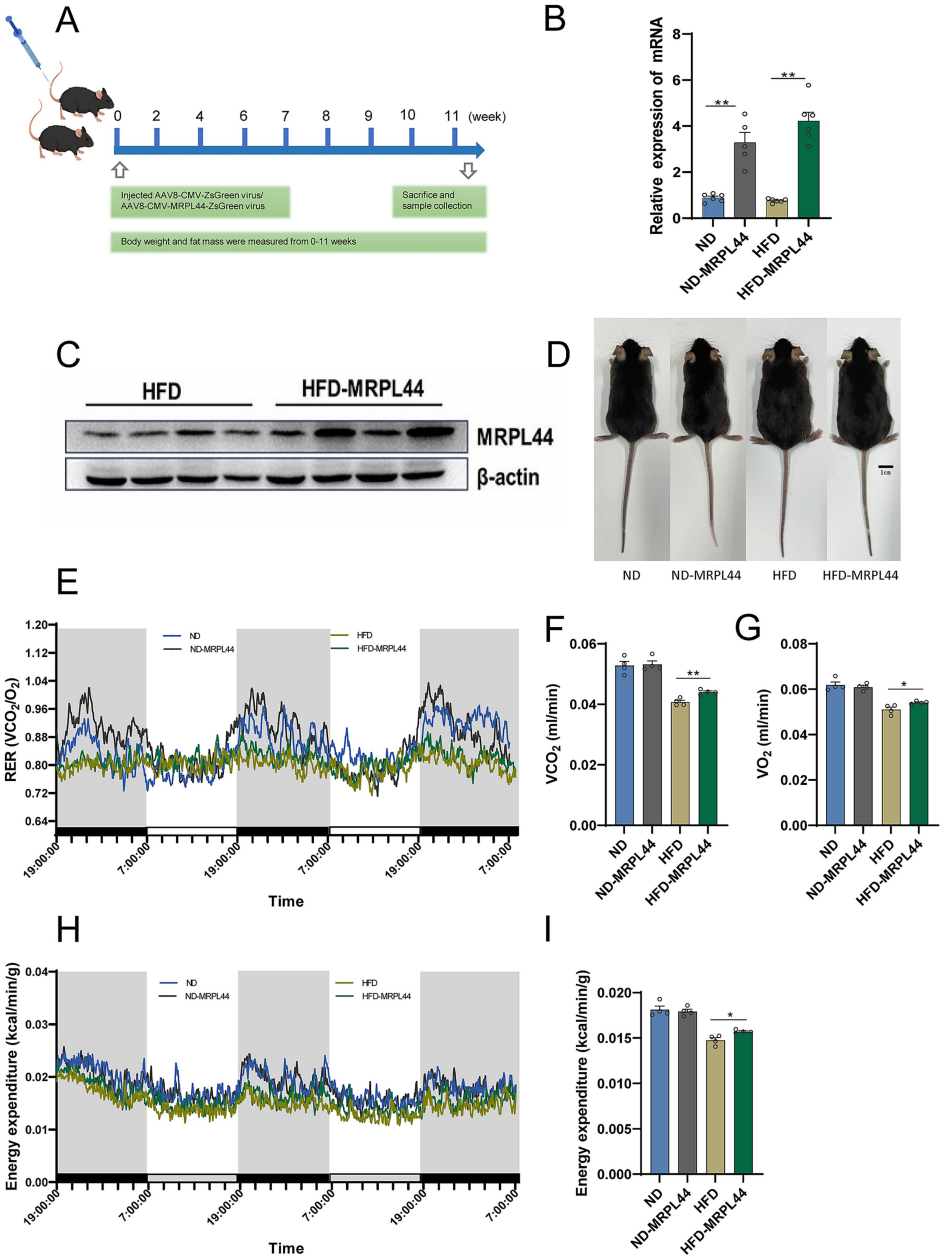
Effect of liver-specific overexpression of MRPL44 on energy metabolism in mice. **(A)** Mice model schematic. **(B)** The expression of MRPL44 mRNA levels. **(C)** Western blot analysis of MRPL44 protein expression in liver tissues from HFD and HFD-MRPL44 groups. **(D)** Representative photographs of mice from ND, ND-MRPL44, HFD, and HFD-MRPL44 groups at the end of the experiment. Scale bar: 1 cm. **(E)** Respiratory exchange rate. **(F)** O_2_ consumption. **(G)** CO_2_ production. **(H)** Energy expenditure line graphs. **(I)** Energy expenditure bar graphs. The mice were 20 weeks, with a sample size of *n* = 4. Data are represented as means ± SEM.

Given the effects of MRPL44 on lipid metabolism, we employed mice with liver-specific overexpression of MRPL44 to examine its role *in vivo*. A schematic diagram of mouse model rearing is shown in Figure 6A. The mRNA and protein levels of MRPL44 were detected in the liver, confirming high upregulation of MRPL44 (Figures 6B,C; Supplementary Figure S1D). The liver morphology of the four groups of mice is shown in Figure 6D. Data analysis revealed that liver-specific overexpression of MRPL44 had no significant effect on body weight, fat distribution, fat content, and fat mass in HFD-fed mice (Supplementary Figures S4A–G). Metabolic cage analysis showed that compared to the HFD group, MRPL44 overexpression did not result in significant changes in the respiratory exchange ratio (Figure 6E), but it did increase oxygen consumption (Figure 6F), carbon dioxide production (Figure 6G), and energy expenditure (Figures 6H,I).

**Figure 7:**
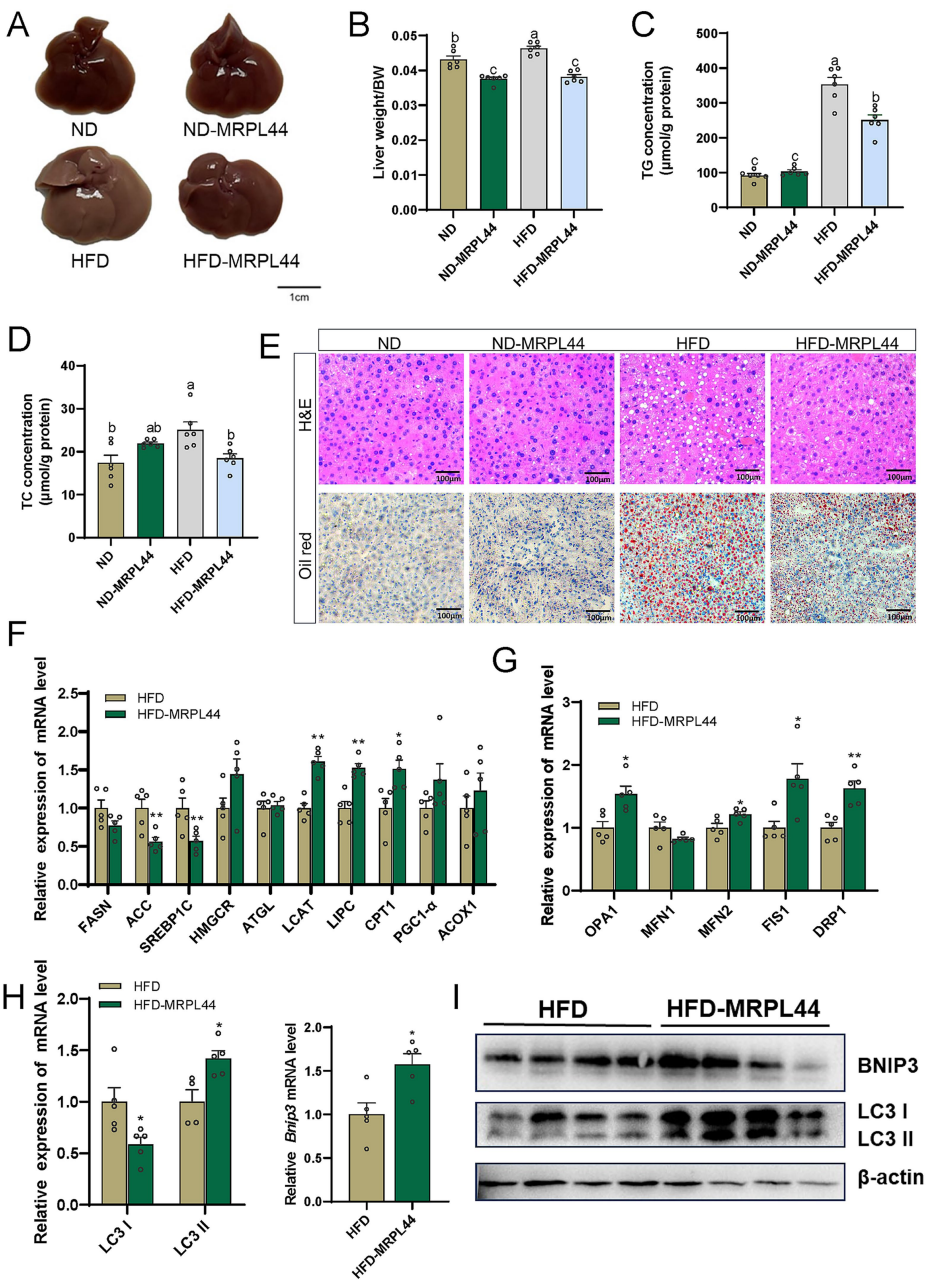
Effect of liver-specific overexpression of MRPL44 on lipid metabolism in mice. **(A)** Representative liver morphology from mice fed a normal diet (ND), normal diet with MRPL44 overexpression (ND-MRPL44), high-fat diet (HFD), or high-fat diet with MRPL44 overexpression (HFD-MRPL44). Scale bar: 1 cm. **(B)** Liver weight-to-body weight (LW/BW) ratio in the four groups. **(C)** Liver TG levels. **(D)** Liver TC levels. **(E)** Representative H&E and oil red o staining of liver sections from the four groups. Scale bar: 100 μm. **(F)** Lipid metabolism-related genes mRNA expression levels. **(G)** Mitochondrial fission and fusion-related genes mRNA expression levels. **(H)** LC3 I and LC3 II gene mRNA expression levels. **(I)** Western blot analysis of BNIP3, LC3I, and LC3II protein expression in liver tissues from HFD and HFD-MRPL44 groups. β-actin was used as a loading control. The mice were 20 weeks, with a sample size of *n* = 3–6. Data are represented as means ± SEM. ^*^p < 0.05 and ^**^p < 0.01.

We then examined liver lipid deposition in the four groups. Liver images showed that MRPL44 overexpression significantly reduced liver weight in HFD-fed mice (Figures 7A,B). MRPL44 also significantly decreased TG (Figure 7C) and TC levels (Figure 7D). However, liver TC levels were increased in ND-MRPL44 mice compared to the ND group. This increase may be attributed to the lower hepatic metabolic load under ND conditions, where MRPL44 overexpression likely promotes cholesterol storage to support metabolic demands. H&E and Oil Red O staining of liver sections demonstrated a marked reduction in lipid deposition in the MRPL44 group (Figure 7E). Subsequently, we quantified the expression of genes related to lipid metabolism and inflammation in the liver of mice. The results indicated that MRPL44 significantly downregulated the mRNA expression of genes involved in fatty acid synthesis and inflammatory factors. Specifically, ACC was downregulated by 44% (*p* = 0.009), SREBP1C by 43% (*p* = 0.018), SCD1 by 66% (*p* = 0.032), TNF-α by 66% (*p* = 0.027), and IL-1β by 69% (*p* < 0.001). Conversely, MRPL44 significantly upregulated the mRNA expression of LCAT by 60% (*p* < 0.001), LIPC by 52% (*p* = 0.001), and CPT1 by 51% (*p* = 0.016) (Figure 7F; Supplementary Figure S1E). In addition, we assessed the expression of genes related to mitochondrial fission, fusion and autophagy. The findings showed that MRPL44 markedly increased the mRNA expression of OPA1 by 0.53-fold (*p* = 0.01), FIS1 by 0.77-fold (*p* = 0.02) and DRP1 by 0.63-fold (*p* = 0.003) (Figure 7G). Moreover, MRPL44 upregulated BNIP3 mRNA levels by 57% (*p* = 0.013), downregulated LC3A by 41% (*p* = 0.028), and upregulated LC3B by 42% (*p* = 0.016) (Figure 7H). Consistent with these changes, protein levels of BNIP3 and LC3 in the liver of MRPL44-overexpressing mice were significantly elevated (Figure 7I; Supplementary Figure S1F).

In summary, these results suggest that MRPL44 mitigates HFD-induced hepatic lipid deposition and improves MASLD.

## Discussion

MRPL44 plays a crucial role in mitochondrial translation, and is closely associated with the progression of hypertrophic cardiomyopathy, highlighting its central role in organelle proteostasis (22). In this study, we extend the biological relevance of MRPL44 to hepatic lipid metabolism. *In vitro*, MRPL44 overexpression significantly reduced OA/PA-induced lipid deposition in HepG2 cells and enhanced mitochondrial function. Consistently, *in vivo* experiments revealed that MRPL44 overexpression attenuated HFD-induced hepatic steatosis in mice. Previous reports have shown that impaired mitochondrial function exacerbates hepatic lipid accumulation in both cultured hepatocytes and NASH animal models, whereas interventions that improve mitochondrial quality or promote fatty acid β-oxidation effectively alleviate steatosis (23). Our findings not only fill a gap in the understanding of MRPL44 within the context of MASLD, but also reinforce the concept that mitochondrial functional integrity is a critical determinant of lipid homeostasis.

Mitochondrial biogenesis involves both fission and fusion processes. An imbalance between these processes can compromise mitochondrial integrity and impair function (24). Mitochondrial fusion and fission are functional determinants of mitochondrial maintenance. Among these, fission-related genes such as DRP1 and FIS1 are critical for balancing mitochondrial quantity and distribution, while fusion-related genes such as MFN1, MFN2, and OPA1 ensure mitochondrial activity (25). The expression of the mitochondrial fusion protein MFN2 is associated with the formation of NAFLD induced by HFD, and liver-specific deletion of MFN2 exacerbates hepatic steatosis and inflammatory factor production in HFD-fed mice (26). Upregulating of MFN2 expression has been shown to counteract lipid deposition and excessive mitochondrial-derived ROS production (27). In this study, we found that MRPL44 overexpression significantly upregulated MFN2 and FIS1 expression (Figures 3C, 7G). Thus, we suggest that MRPL44 may induce mitochondrial fission and fusion, enhance mitochondrial activity, and ameliorate lipid-induced mitochondrial oxidative damage through high expression of MFN2, providing an effective mechanism for MRPL44 to reduce hepatic steatosis.

Fatty acid oxidation primarily occurs in the mitochondria. Dysregulated lipid metabolism can lead to excessive mitochondrial ROS production, disrupting the dynamic balance between free radicals and antioxidant defenses (28). This imbalance alters mitochondrial membrane potential, reduces ATP generation, and ultimately triggers mitochondrial dysfunction (29). Therefore, alleviating mitochondrial dysfunction represents an effective strategy for improving lipid metabolic disorders. For example, apigenin has been reported to reduce fatty acid-induced lipid accumulation in hepatocytes by increasing mitochondrial biogenesis and fusion, decreasing mitochondrial ROS production, and preventing the loss of mitochondrial membrane potential (30). Similarly, our results demonstrated that MRPL44 intervention counteracted OA/PA-induced oxidative damage, enhanced mitochondrial function in HepG2 cells, and reduced ROS production (Figure 4C), additionally, MRPL44 overexpression reversed the loss of mitochondrial membrane potential (Figure 4A), increased ATP production (Figures 3E,I), and elevated mitochondrial OXPHOS activity (Figure 3D,K,L). Collectively, these findings demonstrated the role of MRPL44 in ameliorating mitochondrial dysfunction caused by lipid metabolic disorders. This is consistent with the prevailing view that targeting mitochondria and restoring their function can effectively attenuate lipid accumulation.

Mitophagy is a crucial mechanism for clearing damaged mitochondria under stress and may serve as a protective mechanism in MASLD (31). Impaired mitophagy has been associated with increased lipid accumulation and oxidative stress levels in both HFD-induced obesity models and OA/PA-treated *in vitro* models (32, 33). In line with this, our study suggests that MRPL44 may influence changes in mitophagy. Using the mt-Keima plasmid, an effective tool for visualizing mitophagy under both physiological and pathological conditions, we demonstrated that MRPL44 overexpression significantly enhanced mitophagy. In addition, MRPL44 overexpression increased lysosome numbers and promoted the formation of autophagosomes through coupling between mitochondria and lysosomes (Figure 5C). Analysis of autophagy marker LC3 levels indicated that LC3 mRNA expression was affected in MRPL44-treated cells (Figure 5A; Supplementary Figure S2B). Since the production and degradation of LC3 I and LC3 II are dynamic processes, changes in LC3 II expression at a single time point may not reflect alterations in autophagy. Using autophagy inhibitors such as CQ or bafilomycin A1 is necessary to determine changes in autophagy after intervention. For example, tanshinone I induces autophagy in cervical cancer cells by reducing LC3 I protein levels, and chloroquine treatment significantly increases LC3 I to LC3 II conversion, indicating induced autophagy (34). Similarly, we found that MRPL44 overexpression was associated with decreased LC3 I protein levels and increased LC3 II protein levels. Treatment with chloroquine further increased LC3 II protein levels in the MRPL44-overexpressing group (Figures 5A,B). These results suggest that MRPL44 overexpression promotes autophagy.

Mitophagy involves ubiquitin-dependent and ubiquitin-independent pathways. Receptor proteins containing LC3-interacting regions (LIRs) on the outer mitochondrial membrane interact directly with LC3 to initiate mitophagy (35). Among these, BNIP3 is a well-characterized receptor that facilitates mitophagy by binding to LC3, thereby maintaining mitochondrial quality and cellular homeostasis (36). BNIP3-mediated mitophagy has been implicated in a variety of pathological contexts; for example, activation of BNIP3-dependent mitophagy reduces tissue damage during renal ischemia-reperfusion injury (37), and has also been reported to reduced oxidative stress. In our study, MRPL44 overexpression enhanced BNIP3 expression and promoted their interaction (Figure 5E; Supplementary Figures S2D,E), which was associated with increased mitophagy. Moreover, co-transfection of MRPL44 with siBNIP3 attenuated MRPL44-induced autophagic activity and partially restored triglyceride accumulation (Figure 5G; Supplementary Figures S2E,F). These findings align with previous studies highlighting BNIP3 as a key mediator of lipid homeostasis in hepatocytes (38), further supporting the notion that BNIP3-driven mitophagy enhances fatty acid β-oxidation, alleviates oxidative damage in metabolic disease models. Collectively, we speculate that MRPL44 regulates mitophagy through activation of BNIP3, which in turn alleviates oxidative stress-induced mitochondrial damage and lipid metabolism disorders.

Importantly, MRPs are primarily known for their role in mitochondrial translation and have not previously been implicated in the regulation of mitophagy. The MRPL44-BNIP3 interaction observed in this study may therefore represent a novel extension of mitophagy regulation. In the future, the mitochondrial translational can be linked to the established mitophagy pathways. This connection not only reinforces the protective role of BNIP3 in liver disease, but also broadens the functional relevance of MRPL44 by positioning it as an upstream regulator of mitophagy and hepatic lipid metabolism.

In conclusion, our results demonstrated that MRPL44 promotes BNIP3 expression, prevents fatty acid-induced mitochondrial injury, and enhances mitophagy, thereby maintaining mitochondrial function and ultimately ameliorating hepatic steatosis. These findings provide new insights into the protective role and potential mechanisms of MRPL44 in liver lipid metabolism.

### Limitations of the study

This study has several limitations. First, we used of only male C57BL/6 mice may limited the generalizability of the findings, as numerous studies have reported sex differences in NAFLD susceptibility and mitochondrial quality control pathways. Future studies should include both sexes to better understand these differences. Second, we used only β-actin as the reference gene for qPCR, which is commonly used in NAFLD research. However, the MIQE guidelines recommend validating 2–3 reference genes to ensure accuracy. This limitation will be addressed in future experiments. Lastly, the small sample size in some experiments (three biological replicates per group) may limit statistical power. Future studies should expand the sample size to enhance statistical significance.

## Data Availability

The original contributions presented in the study are included in the article/Supplementary material, further inquiries can be directed to the corresponding authors.

## Glossary

ATP: Adenosine triphosphate
CQ: Chloroquine
DMEM: Dulbecco’s modified Eagle’s medium
GPx: Glutathione peroxidase
HFD: High-fat diet
LPS: Lipase
MASLD: Metabolic dysfunction-associated steatotic liver disease
MDA: Malondialdehyde
MMP: Mitochondrial membrane potential
mtDNA: Mitochondrial DNA
MRPs: Mitochondrial ribosomal proteins
NAFLD: Non-alcoholic fatty liver disease
ND: Normal chow diet
OA: Oleic acid
OCR: Oxygen consumption rate
PA: Palmitic acid
PAE: Predicted aligned error
PMSF: Phenylmethanesulfonyl fluoride
qPCR: Quantitative real-time polymerase chain reaction
ROS: Reactive oxygen species
SDS-PAGE: Sodium dodecyl-sulfate polyacrylamide gel electrophoresis
SOD: Superoxide dismutase
TC: Total cholesterol
TG: Triglyceride
