# Supplementary material for: MRPL44 regulates lipid metabolism in metabolic dysfunction-associated steatotic liver disease through BNIP3-mediated mitophagy
Source: Front Nutr. 2025 Oct 2;12:1662882. doi: 10.3389/fnut.2025.1662882 (PMC12527874; doi:10.3389/fnut.2025.1662882)
Supplement: Supplementary file 1 [file Data_Sheet_1.PDF]

Figure S1

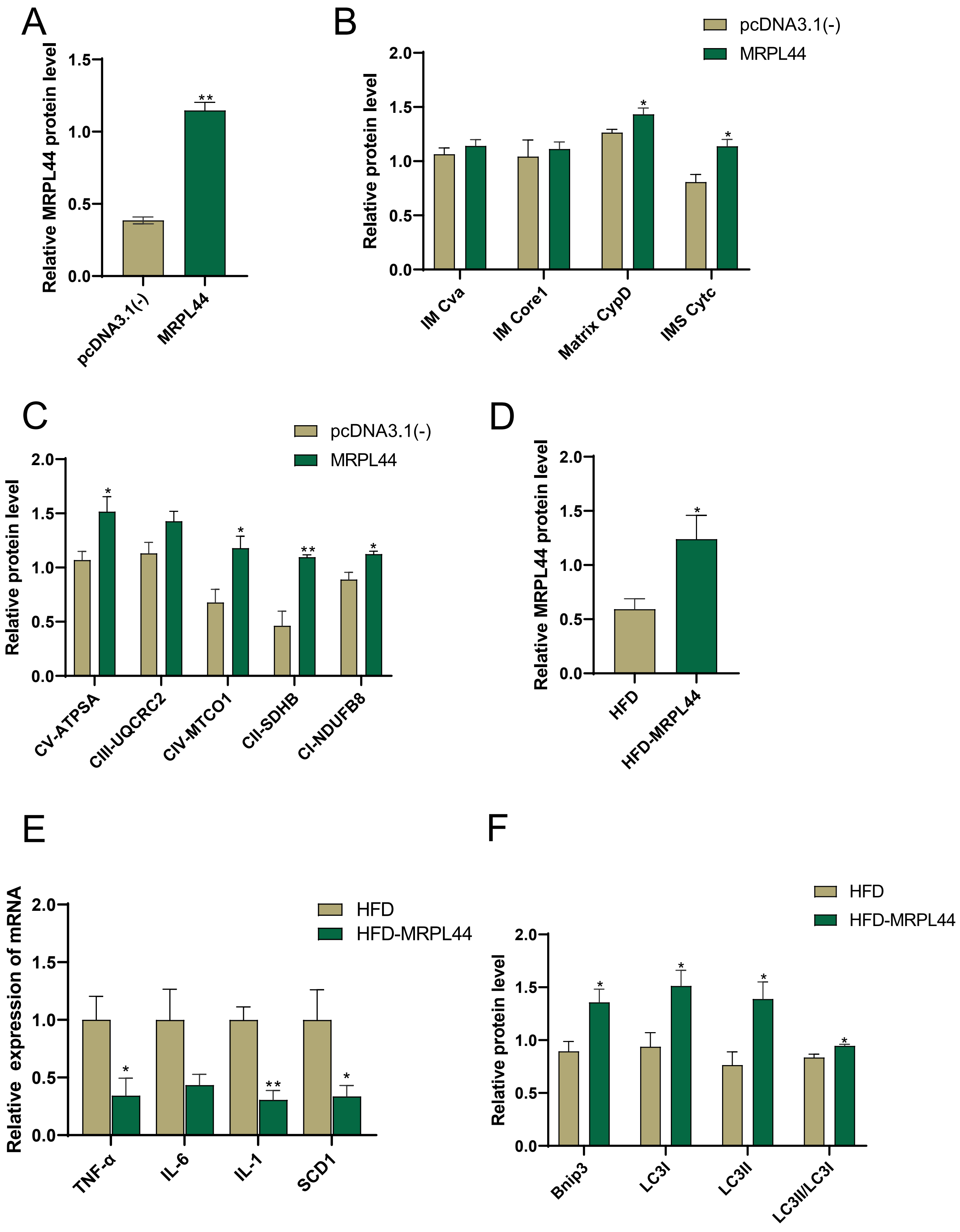

A
Relative MRPL44 protein level
1.5
1.0
0.5
0.0
**
pcDNA3.1(-)
MRPL44
B
pcDNA3.1(-)
MRPL44
Relative protein level
2.0
1.5
1.0
0.5
0.0
*
*
IM Cva
IM Core1
Matrix CypD
IMS Cytc
C
pcDNA3.1(-)
MRPL44
Relative protein level
2.0
1.5
1.0
0.5
0.0
*
*
**
*
CV-ATPSA
CIII-UQCRC2
CIV-MTCO1
CII-SDHB
CI-NDUFB8
D
Relative MRPL44 protein level
2.0
1.5
1.0
0.5
0.0
*
HFD
HFD-MRPL44
E
Relative expression of mRNA
2.0
1.5
1.0
0.5
0.0
HFD
HFD-MRPL44
*
**
*
TNF-α
IL-6
IL-1
SCD1
F
HFD
HFD-MRPL44
Relative protein level
2.0
1.5
1.0
0.5
0.0
*
*
*
*
Bnip3
LC3I
LC3II
LC3II/LC3I

Figure S2

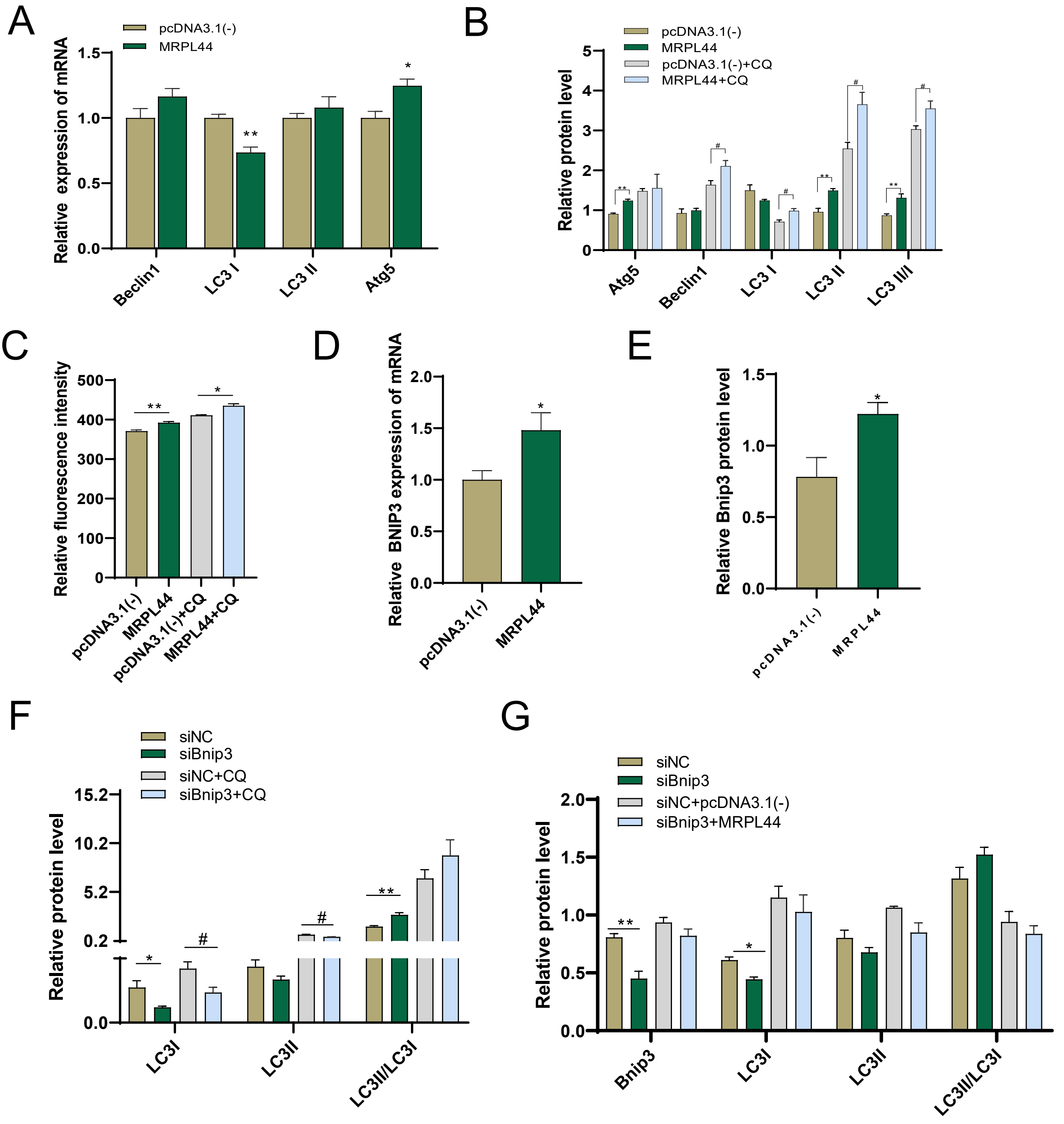
A
pcDNA3.1(-)
MRPL44
Relative expression of mRNA
Beclin1
LC3 I
LC3 II
Atg5
B
pcDNA3.1(-)
MRPL44
pcDNA3.1(-)+CQ
MRPL44+CQ
Relative protein level
Atg5
Beclin1
LC3 I
LC3 II
LC3 II/I
C
Relative fluorescence intensity
pcDNA3.1(-)
MRPL44
pcDNA3.1(-)+CQ
MRPL44+CQ
D
Relative BNIP3 expression of mRNA
pcDNA3.1(-)
MRPL44
E
Relative Bnip3 protein level
pcDNA3.1(-)
MRPL44
F
siNC
siBnip3
siNC+CQ
siBnip3+CQ
Relative protein level
LC3I
LC3II
LC3II/LC3I
G
siNC
siBnip3
siNC+pcDNA3.1(-)
siBnip3+MRPL44
Relative protein level
Bnip3
LC3I
LC3II
LC3II/LC3I

Figure S3

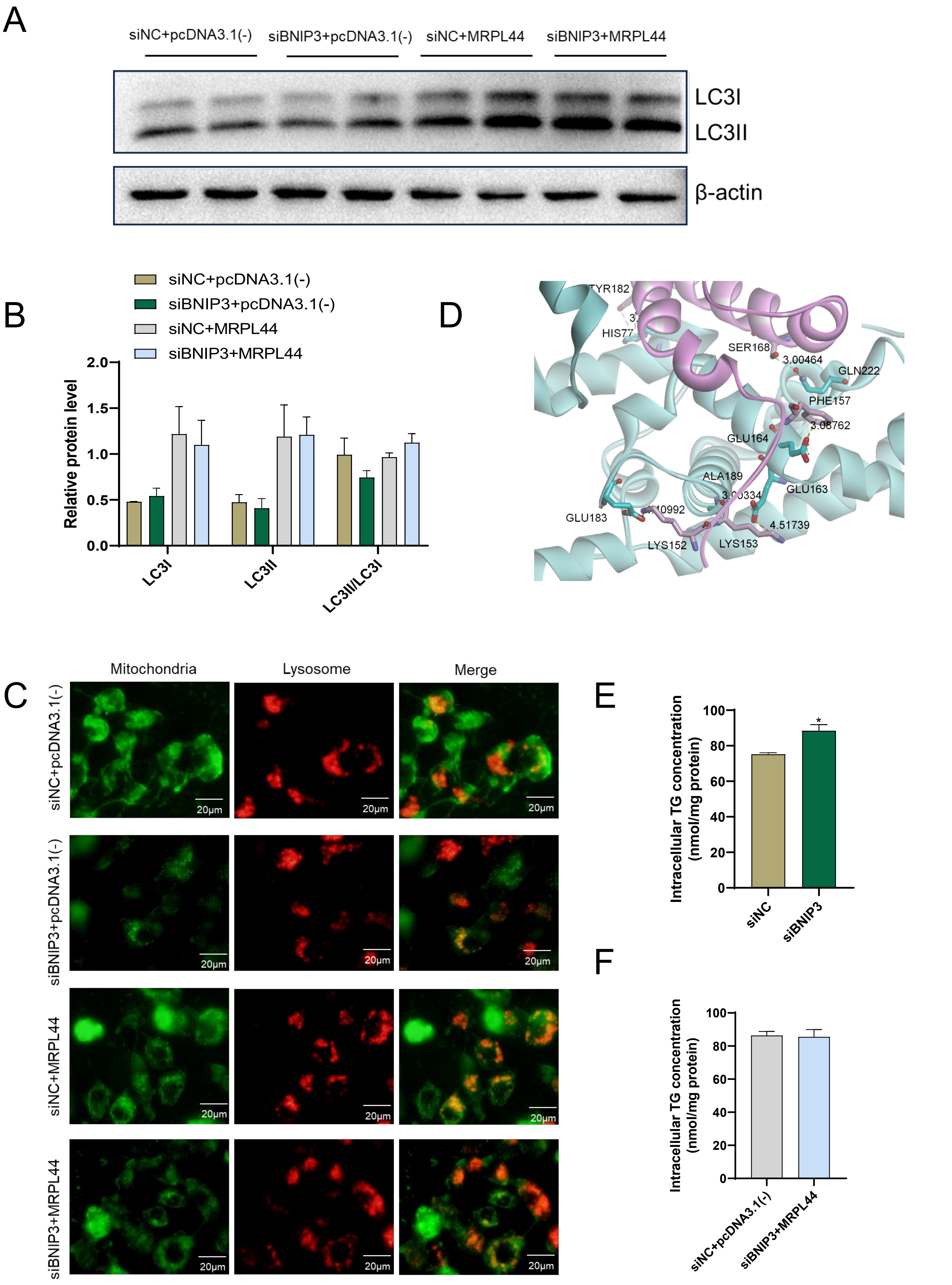
A
siNC+pcDNA3.1(-)
siBNIP3+pcDNA3.1(-)
siNC+MRPL44
siBNIP3+MRPL44
LC3I
LC3II
β-actin
B
siNC+pcDNA3.1(-)
siBNIP3+pcDNA3.1(-)
siNC+MRPL44
siBNIP3+MRPL44
Relative protein level
2.0
1.5
1.0
0.5
0.0
LC3I
LC3II
LC3II/LC3I
D
TYR182
HIS77
SER168
3.00464
GLN222
PHE157
3.08762
GLU164
ALA189
GLU163
GLU183
4.51739
LYS152
LYS153
C
Mitochondria
Lysosome
Merge
siNC+pcDNA3.1(-)
siBNIP3+pcDNA3.1(-)
siNC+MRPL44
siBNIP3+MRPL44
20μm
E
Intracellular TG concentration (nmol/mg protein)
100
80
60
40
20
0
*
siNC
siBNIP3
F
Intracellular TG concentration (nmol/mg protein)
100
80
60
40
20
0
siNC+pcDNA3.1(-)
siBNIP3+MRPL44

Figure S4

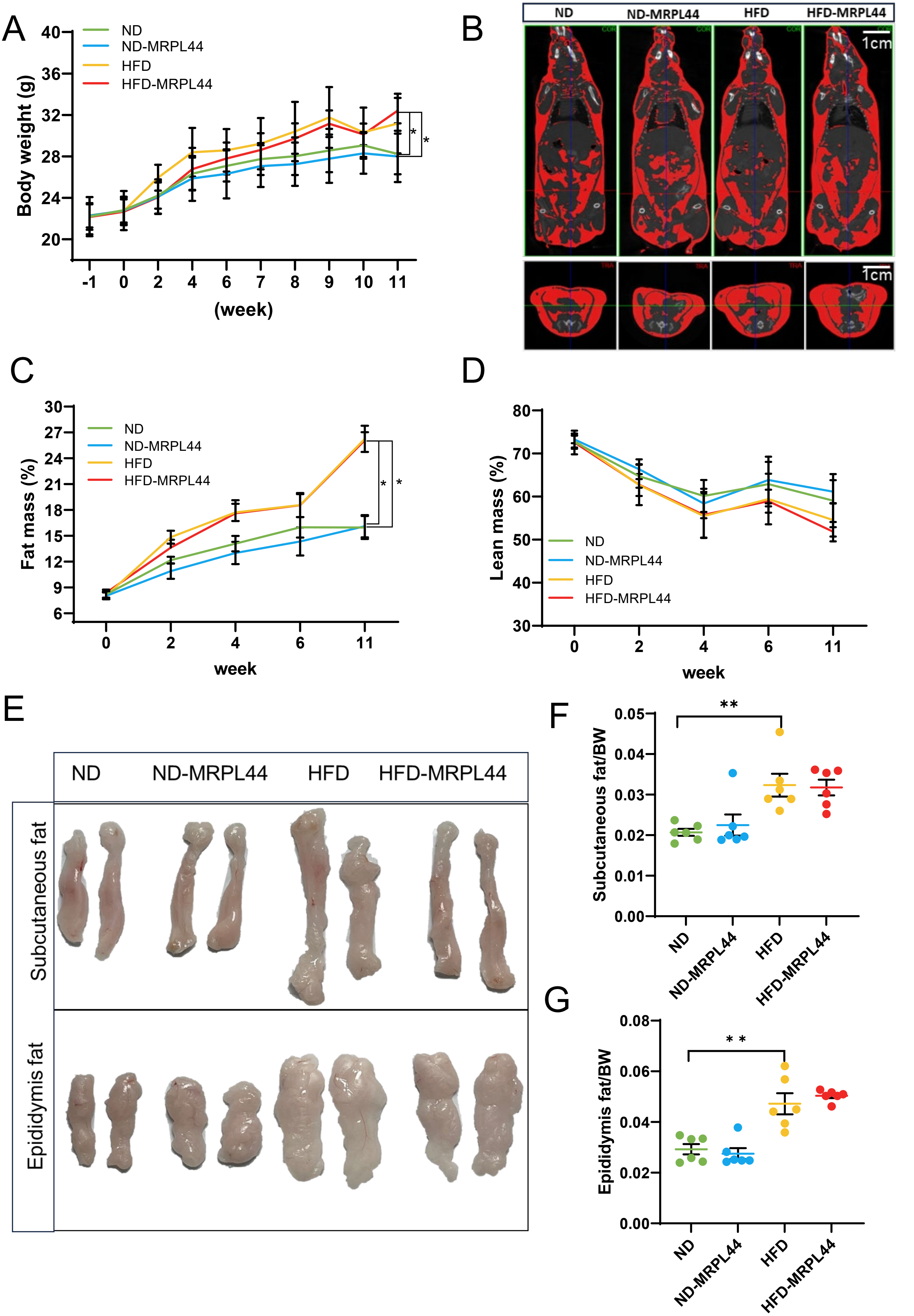
A
Body weight (g)
(week)
ND
ND-MRPL44
HFD
HFD-MRPL44
B
ND
ND-MRPL44
HFD
HFD-MRPL44
1cm
C
Fat mass (%)
week
D
Lean mass (%)
week
E
ND
ND-MRPL44
HFD
HFD-MRPL44
Subcutaneous fat
Epididymis fat
F
Subcutaneous fat/BW
G
Epididymis fat/BW

Figure Legends

Figure S1. MRPL44 expression and its effects on mitochondrial associated proteins in vitro and in vivo models.

(A) The relative protein levels of MRPL44 were quantified by analyzing the gray values of Figure 2B western blot. (B) The relative protein levels of mitochondrial components were quantified by analyzing the gray values of Figure 3K western blot. (C) The relative protein levels of mitochondrial respiratory chain complex subunits were quantified by analyzing the gray values of Figure 3L western blot. (D) The relative protein levels of MRPL44 in liver tissues from HFD and HFD-MRPL44 groups were quantified by analyzing the gray values of Figure 6C western blot. (E) Relative mRNA expression of inflammatory cytokines (TNF-α, IL-6, IL-1β) and lipid metabolism-related gene (SCD1). (F) The relative protein levels in liver tissues from HFD and HFD-MRPL44 groups were quantified by analyzing the gray values of Figure 7I western blot. The mice were 20 weeks, with a sample size of n=3-6. Data are represented as means ± SEM. * $p < 0.05$, ** $p < 0.01$.

Figure S2. MRPL44 enhances autophagy and regulates BNIP3 expression in vitro.

(A) Relative mRNA expression levels of autophagy-related genes. (B) The relative protein levels of autophagy markers were quantified by analyzing the gray values of Figure 5A western blot. (C) Relative fluorescence intensity of LC3 were quantified by Figure 5B. (D) Quantification of BNIP3 mRNA expression. (E) The relative protein levels of BNIP3 in cells were quantified by analyzing the gray values of Figure 5E western blot. (F) The relative protein levels were quantified by analyzing the gray values of Figure 5F western blot. (G) The relative protein levels were quantified by analyzing the gray values of Figure 5G western blot. Data are represented as means ± SEM. * $p < 0.05$, # $p < 0.05$, ** $p < 0.01$.

Figure S3. MRPL44 regulates mitophagy through BNIP3.

(A) Western blot analysis of LC3 proteins. β-actin was used as a loading control. (B) The relative protein levels of LC3 were quantified by analyzing the gray values of Figure S3A. (C) Molecular docking analysis of MRPL44–BNIP3 interaction showing

key binding residues and intermolecular contacts. (D) Representative immunofluorescence images of mitochondrial (green) and lysosomal (red) staining, with merged panels showing mitochondrial/lysosomal co-localization (scale bar = 20 μm). (E-F) Intracellular TG concentration in cells. Data are represented as means ± SEM. * $p < 0.05$.

Figure S4. Effect of liver-specific overexpression of MRPL44 on adiposity in mice. (A) Mice body weight. (B) Fat distribution in mice. (C) Fat mass. (D) Lean mass. (E) Subcutaneous and epididymal fat in mice. (F) Subcutaneous fat weight in mice. (G) Epididymal fat weight in mice. The mice were 20 weeks, with a sample size of n=6-8. Data are represented as means ± SEM. * $p < 0.05$, ** $p < 0.01$.

Supplementary Tables

Table 1. The sequences of MRPL44.

MRPL44	5'-3'
MRPL44-F	ATGGCGTCCGGGCTGGTAA
MRPL44-R	CTAGCTGGCAGTGATGCTCTTT
infusion-MRPL44-F	GATATCTGCAGAATTATGGCGTCCGGGCTGGTAAGA
infusion-MRPL44-R	CAGTGTGGTGGAATTCTAGCTGGCAGTGATGCTCTTTT
siRNA1	GGATTACCATGCAGAAATA
siRNA2	GGACATCGGTTACAGGAAA
siRNA3	GGGACATCTTTTTCACAGA
Negative control	UUCUCCGAACGUGUCACGUTT

Table 2. Primer sequences for real-time PCR amplification.

Genes	Forward	Reverse
MRPL44	TCCAGAAGGAGTTAGAGCGGCA	GGAAAAGTTTTCCTGTAACCGATG
FASN	GGTTCTGGTTGCCTTGGTAGGA	CTGTGTGCATCTGGCTGGTAGA
ACC	TTCACTCCACCTTGTCAGCGGA	GTCAGAGAAGCAGCCCATCACT
SREBP1C	ACTTCTGGAGGCATCGCAAGCA	AGGTTCCAGAGGAGGCTACAAG
HMGR	GACGTGAACCTATGCTGGTCAG	GGTATCTGTTTCAGCCACTAAGG
LCAT	TGGCTCCTCAATGTGCTCTTCC	CACCACATCTGGTTTGTCCAGC
CPT1	GATCCTGGACAATACCTCGGAG	CTCCACAGCATCAAGAGACTGC
LIPC	TCCATCGGTGGAACGCACAAGA	GCGTAAAGGTATGAATGGCATCC
HSL	AGCCTTCTGGAACATCACCGAG	TCGGCAGTCAGTGGCATCTCAA
FIS1	GCTGGTGTCTGTGGAGGACC	GCAGCACGATGCCTTTACG
DRP1	TAGCTACGGTGAACCCGTGGA	ACCACCGCATAGCTCCGAAGT
MFN1	GGTGAATGAGCGGCTTCCAAG	TCCTCCACCAAGAAATGCAGGC
MFN2	ATTGCAGAGGCGGTTCGACTCA	TTCAGTCGGTCTTGCCGCTCTT
OPA1	GTGGTTGGAGATCAGAGTGCTG	GAGGACCTTCACTCAGAGTCAC
TNF-α	CTCTTCTGCCTGCTGCACTTTG	ATGGGCTACAGGCTTGTCACTC
IL-1	CCACAGACCTTCCAGGAGAATG	GTGCAGTTCAGTGAGTGATCGTACAGG
IL-6	AGACAGCCACTCACCTCTTCAG	TTCTGCCAGTGCCTCTTTGCTG
SCD1	CCTGGTTTCACTTGGAGCTGTG	TGTGGTGAAGTTGATGTGCCAGC
BECLIN1	CTGGACACTCAGCTCAACGTCA	CTCTAGTGCCAGCTCCTTTAGC
LC3A	GCTACAAGGGTGGAGAGCAGCT	CTGGTTCACCAGCAGGAAGAAG

LC3B	GAGAAGCAGCTTCCTGTTCTGG	GTGTCCGTTCACCAACAGGAAG
ATG5	GCAGATGGACAGTTGCACACAC	GAGGTGTTTCCAACATTGGCTCA
ACOX1	GGCGCATACATGAAGGAGACCT	AGGTGAAAGCCTTCAGTCCAGC
PGC1-α	CCAAAGGATGCGCTCTCGTTCA	CGTGCTTGTAGTGGCTTGACT
β-acin	CGACAGGATGCAGAAGGAGAT	CAAGAAAGGGTGTAACGCAACTA
28srDNA	AGGACCCGAAAGATGGTGAACTA	CGGAGGGAACCAGCTACTAGAT
ComplexII	CAAACCTACGCCAAAATCCA	GAAATGAATGAGCCTACAGA
